# Domestic Violence During Pregnancy in Greece

**DOI:** 10.3390/ijerph16214222

**Published:** 2019-10-31

**Authors:** Evangelia Antoniou, Georgios Iatrakis

**Affiliations:** Department of Midwifery, University of West Attica, Athens 12243, Greece; giatrakis@uniwa.gr

**Keywords:** violence, pregnancy, partner, risk factors

## Abstract

There are no data about the prevalence of domestic violence during pregnancy in Greece. The purpose of this study is to determine the prevalence and the associated factors of domestic violence in a representative population of pregnant women in Greece. Five hundred and forty-six consecutive women, in outpatient clinics of two Public General Regional Hospitals in Athens, agreed to answer anonymously the Abuse Assessment Screen (AAS) questionnaire, translated into the Greek language. Five hundred and forty-six questionnaires were returned (100% response rate), revealing that the prevalence of domestic violence in pregnancy is 6%, with 3.4% of the sample having being abused since the beginning of pregnancy, mainly by their husband/partner. The factors associated with higher risk of abuse during pregnancy were nationality, socio-economic background, and educational level. Foreign women or women with a foreign partner, unemployed individuals, housewives, and university students faced a higher risk of being abused. A substantial age difference (≥10 years) in the couple, a history of abortions, and an undesired pregnancy also increased the risk of violence in pregnancy. These findings suggest that prenatal care is an important period for discussing abuse and, in the end, encouraging women to seek help.

## 1. Introduction

According to the Council of Europe declaration, domestic violence is “the violence occurring in the family or domestic unit, including, inter alia, physical and mental aggression, emotional and psychological abuse, rape and sexual abuse, incest, marital rape, or rape between regular or occasional partners and cohabitants, crimes committed in the name of honor, female genital and sexual mutilation, and other traditional practices harmful to women, such as forced marriages” [1]. The term ‘domestic violence’ is used in many countries to refer to partner violence but the term can also encompass child or elder abuse, or abuse by any member of a household. Intimate partner violence is a more specific term defining one of the most common forms of violence against women which includes physical, sexual, and emotional abuse and controlling behaviors specifically by an intimate partner [2].

### 1.1. Prevalence of IPV in Greece

A survey [3] was carried out in 2003 with the aim of recording the nature and magnitude of domestic violence in Greece. In a sample of 1200 women, aged 18–60, 61.9% of women living in semi-urban centers stated that they frequently experience verbal or psychological abuse by their husband/partner. The percentage was slightly lower for women living in urban centers and agricultural areas. Furthermore, 5.3% of the women in semi-urban centers, 3.7% in urban and 3% in agricultural areas stated that they have been victims of physical violence. Finally, 3.3%–4% of women stated that they are coerced into having sexual intercourse. These results constituted the leverage for the implementation of political, social, and legislative measures to deal with violence against women in the family [4]. A similar study in Greece concluded that self-esteem, experience of abuse during childhood, gender, age, and years of cohabitation correlated with an increased risk of specific forms of intimate partner violence. Furthermore, the occurrence of one form was likely to be related to other forms of violent behavior [5].

The disconcerting results of the 2003 nation-wide survey made it clear that it was necessary and urgent to explore domestic violence against pregnant women, who are particularly vulnerable due to increase in their physical, social, emotional, and financial needs during pregnancy.

### 1.2. Global Prevalence of IPV in Pregnancy

According to the study by Tailleu and Brownridge [6], the landmark publication on the prevalence of violence in pregnancy is that by Gazmararian et al. [7], which reviews studies to-date on the phenomenon of violence in pregnancy. This article found that in developed countries the rates of violence in pregnancy ranged to a great extent from 0.9% to 20.6% whereas the majority [8,9,10,11,12,13] ranged between 3.09% and 8.3%. The survey methodology was a key factor that determined the deviation of the findings. According to a more systematic and detailed study of the surveys the higher percentages are linked to: (a) More inclusive definitions of abuse; (b) more than a single item asking about abuse experiences; (c) multiple interviews for each person; (d) adolescence; (e) respondent’s low income [7].

The surveys that followed concerned both the developed and the less developed countries and the various types of violence (physical, sexual, emotional, and verbal) while in developing countries, it became obvious that intimate partner violence during pregnancy negatively affects obstetric-related outcomes [14] and women’s mental health in the postnatal period [15]. Moreover, the percentages of physical violence ranged between 0.9% and 30% while [16,17,18,19,20,21,22] certain surveys measuring physical violence separately found percentages ranging from 3.0% to 10.9%.

### 1.3. IPV in Pregnancy: Risk Factors

Higher percentages of prevalence of the IPV in pregnancy are linked to younger age, lower income, and unmarried women [18] while cultural differences between developed and non-developed countries in matters of violence may account for the percentage differences in relation to violence before and during pregnancy. An intercultural survey has shown that the degree of social and economic inequalities between the two genders in a society affects the percentages of violence in the general population of women [23] and may also be a factor for violence in pregnancy. However, there is a strong debate on how gender equality in the wider society affects gender-based violence (GBV) against women, including varieties of patriarchy in different societies [24]. With regard to the victim’s characteristics, certain risk factors such as history of violence seem to correlate with higher rates of violence in pregnancy [18,21,25], followed by ethnicity, with higher percentages of victims in women belonging to minority groups [21,26], particularly individuals with lower levels of education and of lower socioeconomic status [23,27], substance abuse [19,26,27,28,29], and, finally, adolescence [11]. In terms of the key characteristics of the perpetrator as for risk factors increasing the incidence of violence in pregnancy, exercise of power and control [30] and substance abuse [12,13] are the main reasons, as shown in relevant studies, for violence by women’s partners.

A third group of factors linked to the increase of violence in pregnancy is related to the circumstances and conditions of pregnancy. Undesirable pregnancy and accidental or unplanned pregnancy constitute the most important factors of violence in pregnancy [31,32].

### 1.4. Consequences of IPV in Pregnancy

The exercise of violence in pregnancy has disastrous consequences both for pregnant women and fetuses, such as recurrent miscarriages, hemorrhage, preterm rupture of membranes, premature birth, premature placental abruption, and low birth weight [11,33,34,35]. Other consequences are stress, addictions, suicide attempts, depression, and obstetric and gynecological complications especially for the pregnant women [16,36,37]. Female victims of domestic violence are isolated and usually suffer from depression, eating disorders, panic attacks, and anxiety. They also harm themselves and the fetus by alcohol, drug abuse, or medicines [27,28,38].

Women, when victims of domestic violence, have special needs and they should have personalized care. International surveys have suggested that women who were victims of family violence used less healthcare services and delayed prenatal care [39]. Midwives and any health professionals responsible for the care of pregnant women should cooperate with them and try to understand if the reason of certain behaviors (i.e., psychological problems, drug use, and substance abuse) is due to cases of domestic violence. The purpose of this study is to raise midwives’ and doctors’ awareness on the signs veiling intimate partner violence during pregnancy.

## 2. Methodology

### Study Design, Data, and Sampling

The aim of this survey is to statistically study the prevalence of violence in pregnancy in Greece. The demographic data of pregnant women who are victims of violence and of their perpetrators and the frequency and types of violence were quantified and evaluated in relation to possible co-dependent variables.

The sample consisted of 546 pregnant women (470 Greek and 70 foreign women (according to their statement about nationality in the questionnaire)). The women included in the study were recruited by convenience at the outpatients’ clinics of “Alexandra” (*N* = 264) and “Elena Venizelou” (*N* = 282), two Public General Regional Hospitals in Athens, on the days when the investigator-midwife was there to administer the questionnaire in the period from August 2009–September 2009. The sample is taken from two large public hospitals with the highest number of births per year. Women who are examined in the obstetric clinics come from all over Greece. Pregnant women participating in the study were on average 32.95 weeks along in their pregnancy with a standard deviation of 6.78 weeks; so, they were mostly in relatively advanced stages in their pregnancy at the time of the study. All women accepted to fill in an anonymous questionnaire in a private space provided at the outpatients’ clinics after being fully informed and after signing a consent form for their participation in this survey in accordance with the rules of research, bio-ethics and professional conduct. No woman was accompanied by her partner/husband.

The questionnaire was translated into the Greek language and Abuse Assessment Screen (AAS) weighted by the investigator-midwife. The researcher has translated into Greek and weighted the AAS questionnaire as an undergraduate thesis at the National School of Public Health. The process of translation into Greek was done in accordance with the procedure proposed by the Trust Scientific Advisory Committee (1997) and followed by cultural adaptation and pilot implementation. The Abuse Assessment Screen (AAS) questionnaire is the oldest screening tool for domestic violence that includes five questions, three of which yield results similar to those given by controlled and approved research tools. To compare the three questionnaire questions in the methodology of the work of MacFarlane et al. (1992), the 30-question ISA was used, and ISA-P and ISA-NP sub-scales were designed for women classified as abused or non-abused. The answers to the first three questions either confirm or reject the occurrence of violence in pregnancy. The basis in this survey for the separation of abused and non-abused women, according to the methodology of MacFarlane et al. (1992) [40], was that the abused women were the ones that had answered affirmatively to one of said three questions, i.e., 2, 3, 4, of AAS also analytically includes parts of the body to show injuries. We used the HITS tool (Hurt, Insult, Threat, Scream) to control our results. Its four questions are graded based on the items of the Likert 5-point scale. Mark higher than or equal to 10 is taken as affirmative. The sensitivity of HITS is 96% and the specificity 91% [41].

The statistics program SPSS 18.0 (SPSS Inc., Chicago, IL, USA) was used for the statistical data analysis.

A multivariate analysis was not used since the response variable we were interested to analyze was only one. Moreover, a multiple analysis model was also not preferred since the sample size was relatively small to use maximum likelihood-based statistical checks (e.g., generalized least square models). Instead, the “Fisher’s Exact Test (in tandem with the confounding factor analysis) was deemed more appropriate for its increased validity in such cases and it was used to investigate statistical correlations between categorical variables to achieve the highest possible accuracy in the procedure of analysis and drawing conclusions. Type of injuries and other characteristics of violence during pregnancy are included in Table 1.

Continuous variables were summarized using the mean value and standard deviation (±SD), while for nominal and ordinal variables, Tables of frequencies and relevant frequencies were used. To correlate said variables with the sample’s characteristics, the statistical hypothesis testing was used, with 5% (*p*-value < 0.05) as statistical significance level. The answers with the total grades for pregnant women in the AAS questionnaire were used as dependent variables to draw conclusions related to the objective targets of the survey. The questions related to the social-demographic data of the pregnant women and their partners were taken as independent variables. A confounding factor analysis has been conducted and the results presented in Table 2 illustrate the relationships between domestic abuse and associated factors after having controlled for confounding effects of other variables including women’s nationality, partner’s nationality, educational level, and partner’s level of agreement to the continuation of the pregnancy.

The following permits were taken as shown below:

Athens General Hospital “Alexandra”, Scientific Board, 5th Meeting on 1.07.09, Item 29, Approval of Evangelia Antoniou conducting a research using a questionnaire; Athens General Maternity Hospital “Elena Venizelou”, Scientific Board, Extraordinary Meeting on 30 June 2009, Ref. No. 182, Approval of Evangelia Antoniou conducting a research named “Domestic Violence and Pregnancy”. Consent forms were also given to all women participating in the study, which were duly signed by them, under the provision that no names, photographs, or other personal data would be used for their identification.

No other permits or licenses were required under the national law, given the fact that the present research took place in 2009, that is much earlier than the establishment of the legislation of the Ethical and Deontology Committee for Research of the Panteio University of Social and Political Sciences (Article 21 of 4521 law, 2018). Therefore, no ethical approval was required and thus not provided to a corresponding committee.

## 3. Results

### 3.1. Prevalence of IPV in Pregnancy

All pregnant women (546) we approached accepted voluntarily to participate in the survey and, therefore, the analysis was carried out for 100% of the answers.

Based on the three questions to confirm abuse in pregnancy, 6% (33 participants) answered that they have been abused; 3.4% (18 participants) answered that they have been abused (by their partner) since the beginning of pregnancy. We correlated the HITS and AAS results and found out that both tools show satisfactory percentages of agreement in their results.

The most frequent part of injury is the face (3.1%—17 participants), followed by the abdomen (1.3%—7 participants); 4.8% (26 participants) of the abused pregnant women stated that no part of their bodies was injured. Type of injuries and other characteristics of violence during pregnancy are included in Table 1.

Regarding the means of violence, the most common one is slapping or pushing for 2.7% (15 participants) of the abused pregnant women, followed by punching, kicking etc. for 1.5% (8 participants).

### 3.2. Factors Associated with High Risk of IPV Pregnancy

Factors related to violence in pregnancy are listed in Table 2. Nationality seems to differentiate women at a statistically significant degree regarding abuse. Only 5.2% of the Greek women in our sample were abused; the corresponding percentage for women of other nationalities was almost 3.5 times higher (17.9%). Furthermore, the partner’s nationality plays a significant role as a factor of abuse only for Greek women and it also seems that choosing a foreign partner increases the possibilities of abuse by 3.5 times (5.5% vs. 18.9%). Greek women with a foreign partner have higher possibility by 4.5 times to be abused vs. the ones with Greek partners while foreign women generally report very high percentages of abuse irrespective of their partner’s nationality.

Although no statistically significant correlation between abuse and level of education is observed, it is obvious that abuse is decreased at higher educational levels (universities, post-graduate and PhD levels), irrespective of nationality. The percentage of abused women in the population of Greek women who are elementary or junior high school graduates is 15%, for secondary education graduates it is 6.9%, and for higher education graduates, the percentage is 4.4%, whereas for Greek women who are holders of a post-graduate or doctoral degree, the abuse percentages are 1.4%.

It seems that there is a statistically significant relation between the profession of a woman and the possibility of abuse. For Greek women, the higher abuse percentages are reported in the “unemployed women” (15.4%), “housewives” (7.1%), and “university students” (20%) population. No abused woman was found in the self-employed population. In the case of foreign women, given that housework, unemployment and private sector employee concerns almost 80% of the foreign women sample, no differentiation seems to exist regarding their work.

Regarding marital status, married women are less abused; specifically, by 78.8% vs. single women (25% abused single women and 5.3% married women).

Regarding the woman’s age, there is no statistically significant relation between the percentages of abuse in the various age groups. However, it seems that the substantial age difference in a couple strengthens abuse percentages. The chi-square test demonstrated that there is a statistically significant correlation between the couple’s age difference and the degree of abuse. Actually, it seems that the percentage of abuse increases proportionally to the difference of the absolute age.

A history of abortions seems to be related to the occurrence of abuse. The percentage of abuse is doubled in women having undergone abortion vs. women that have never undergone such a procedure (12% vs. 5.3%). The possibility of abuse in cases that the partner is in favor of pregnancy termination is almost 7 times higher vs. the cases that the partners of women wanted the pregnancy (36.4% vs. 5.5).

Whether the tests proposed by physicians or midwives are performed or not is very strongly linked to abuse. The percentages of abuse in women that do not have all the tests is more than 12 times higher compared to women that had all the range of tests performed (54.5% vs. 4.6%), a result that could be explained by the fact that foreign and less educated women are more likely to suffer from gender based violence.

### 3.3. Action Taken

After the questionnaire was filled, information about women’s shelters and/or services for abused women were given to the women included in the study.

## 4. Discussion

The results of this survey demonstrate that violence in pregnancy occurs also in Greece. According to our findings, 6% of pregnant women are abused by their partners (Table 1); this percentage falls within the range (0.9%–22.0%) given by other relevant surveys in other European and N. America countries [7].

The percentage of violence is slightly higher than the lowest limits of the percentage in the general women’s population, as documented in the first epidemiology study of domestic violence in Greece (3%–5.3%).

The perpetrator reported was the partner (61.1%) and the most commonly injured part of the body was the face (3.1%), followed by the abdominal area (1.3%), a fact which shows aggressiveness, particularly threatening to the fetus. It is impressive that 4.8% report no injury to a specific part of their body, which leads to the conclusion that slapping or pushing, reported as the most common type of violence by 2.7% of abused pregnant women, did not leave any mark or was not considered causing injury to a specific part of the body. On the contrary, 1.5% of the sample reported punching, kicking etc. as a form of violence which agrees with the 1.3% that reported injuries caused at specific parts of the body (Table 1).

We also observe that even during pregnancy, women are afraid, i.e., they suffer psychological violence (2.8%), while coercion to sexual intercourse is slightly more restricted (1.9%), especially if we compare it to the corresponding percentage in the general population of Greek women (3.3%–4%).

With regard to the factors relating to violence in pregnancy, it seems that nationality plays a significant role as foreign pregnant women are abused 3.5 times more frequently than Greek pregnant women (17.9% vs. 5.2%). It is a finding agreeing with the findings of other studies in the literature review [19,26]. Nationality is a key factor of abusive behavior in pregnancy, found in a latent form in the correlation between abuse and religion and abuse and work; this leads to the assumption that the special living conditions of immigrant women in Greece increase the incidence of violence in pregnancy. The customs and experiences that immigrant women carry from their country of origin may play a significant role in their abuse even if they live in a different social framework. Another interpretation could be that women belonging to national minorities are more willing to report violence compared to the domestic population [42].

Another factor interacting with violence in pregnancy is the educational level. Low educational level increases the possibility of violent behaviors in pregnancy (Table 2). This finding agrees with the data of abuse in the general women’s population in Greece, where physical and psychological/verbal abuse is mainly inflicted on women with elementary partial education, but also with the findings from surveys in other countries.

Pertaining to correlating work and violence in pregnancy, it was proven that women involved in housework, unemployed or university students are more likely to be submissive to abusive partners (7.1%, 15.4%, and 20% respectively). Financial insecurity and dependence on the partner obviously agrees with the general submissive attitude. However, nationality was proven again to be a latent factor defining the relation between work and abuse, as very few working foreign women were included in the sample (20) and only one working as a civil servant was abused (Table 2).

Pertaining to the perpetrator’s characteristics, violence in pregnancy seems to be related to the partner’s nationality only for Greek pregnant women, as they have 5 times higher possibility of being abused vs. Greek women with Greek partners (Table 2). The incidence of violence is related to the partner’s nationality as foreign partners tend to be significantly more abusive even towards their Greek partners either in a relationship or marriage.

With regard to the background of the pregnancy and, in particular, conception, previous miscarriages in the life of a pregnant woman are not related to violence in this pregnancy; on the contrary, abortions are related to abuse at a two times higher percentage (12% vs. 5.3%), while pregnancy which is not desired by the partner also increases the percentages of violence by seven times (36.4% vs. 5.5%). It can be assumed that the previous termination of pregnancy may have been the result of physical or psychological violence (Table 2).

Our findings agree with the results of foreign studies that concluded that women suffering violence in pregnancy were more possible to report that their pregnancy was undesirable compared to non-abused women. This is also linked to the fact that abused women do not observe the medical instructions concerning the required tests for pregnancy. Although all women, attend courses for pregnant women, abuse is linked to inadequate care in pregnancy which does not depend on the financial status of the women or her partner but the abusive behavior per se. The percentage of abused women not obeying medical instructions concerning tests was 54% vs. 4.6% of the non-abused (Table 2).

## 5. Strength and Limitations of the Study

This is the first published study about domestic violence of pregnant women in Greece. The sample is representative because it was taken from two large public hospitals with the highest number of births per year, while women who were examined in the obstetric clinics came from all over Greece. Our study was cross-sectional and, therefore, it was impossible to find causal links and draw conclusions. A selection error may also exist as only pregnant women in Public Hospitals were selected. The small number of the sample is another limitation of the study. Furthermore, the results cannot be generalized to another population of pregnant women in private hospitals. Similar limitations characterize other studies related to domestic violence [43]. Furthermore, an effort was made to detect incidents of violence at least once during pregnancy, when the pregnant woman was asked. It was impossible to investigate if there were changes after the questionnaire was filled in by a pregnant woman regarding the kind or intensity of the incidents of violence during pregnancy.

Although a structured questionnaire was used in this study and all principles of privacy were observed, there is the possibility the number of pregnant women that disclosed incidences of violence to be smaller than the number of really abused pregnant women. This happens because we do not know if pregnant women that were abused had a higher or lower possibility for participation in the study although it is much more possible for the abused pregnant women to be under-represented in the sample of pregnant women in our study. Furthermore, in other studies, domestic violence during pregnancy is difficult to be openly admitted as the partner may be present and this is a factor that can decrease the number of pregnant women disclosing incidents of violence [44]. The women in the sample may have hidden incidents of violence trying not to be differentiated from what it is considered socially acceptable. Finally, working as a counselor in a counseling center for women that narrate experiences of intimate partner violence relationships can affect the meaning of intimate partner violence for counselors, resulting in a broader and deeper interpretation and approach toward this phenomenon, a factor not included in our study [45].

## 6. Conclusions

In the present study, we have shown that pregnancy may in certain circumstances lead to violent behaviors. We have seen that certain vulnerable populations, under certain circumstances, are more likely to become victims during pregnancy. Immigrant women in Greece appear to be affected by violence and this is due to two main factors: (a) The cultural characteristics of the place of origin, which sometimes under certain conditions and sometimes in a general and latent or explicit manner, allow the companion to exercise violence and control to the woman, in particular the pregnant woman; and (b) living conditions in the place of destination—in this case Greece—which are extremely precarious and make themselves and their children victims of their partners and their commands. Depending on the place of origin, living conditions and educational level, women may be victims of violence in a sensitive period of their life.

It is necessary to investigate thoroughly the particular forms of violence that women experience in pregnancy, i.e., whether physical violence is the predominant form in relation to other forms just as obnoxious, such as emotional, psychological, or sexual violence.

The crucial question that remains to be answered is the impact of pregnancy as an event that brings about a series of changes in the couple’s dynamics, causing de facto out of control situations that lead to violence against the woman and especially the mother. The profile of the perpetrator and the victim, the couple’s previous dynamics, the circumstances of conception are also factors in investigating the dependence of the “violence-pregnancy” variables.

As violence against women is inextricably linked to the morals and norms of any society, its elimination cannot be an issue cut off from violence as a broader social phenomenon. We expect, therefore, that the emergence of different forms of violence, favored in times of social economic and political crisis, as a way of resolving differences that are contrary to dialogue and mutual respect, will also lead to an increase in violence at the core of society, namely in the family.

Therefore, the policies that need to be designed and implemented to tackle violence in society will be a general framework for promoting human rights values and principles in order to combat inequality and abuse of women of all ages and in particular pregnant women.

## Figures and Tables

**Table 1:** Type of injuries and other characteristics of violence during pregnancy.

Injuries & Violence Characteristics	F	%
Abused in Pregnancy		
Yes	33	6.0%
Abused Since the Beginning of Pregnancy		
Yes	18	3.4%
Most Frequent Part of Injury		
Face	17	3.1%
Head	4	0.7%
Breast	3	0.5%
Back	3	0.5%
Upper Limbs	5	0.9%
Lower Limbs	1	0.2%
Hips	2	0.4%
Abdomen	7	1.3%
Other	1	0.2%
Means of Violence		
Slapping or pushing	15	2.7%
Punching, kicking etc.	8	1.5%
Internal, permanent injury to head	1	0.2%
Use of weapon	1	0.2%
Threat	1	0.2%
Are You Afraid of Your Partner or Someone Else?		
Yes	13	2.8%
Were You Forced to Intercourse in the Previous Year?		
Yes	9	1.9

**Table 2:** Factors related to violence in pregnancy.

Factor	Non-Abused		Abused		Total		*p*-Value ^1^
	*N*	%	*N*	%	*N*	%	
Nationality							0.001
Greek	385	94.80%	21	5.20%	406	100.00%	
Other	55	81.10%	12	17.90%	67	100.00%	
Partner’s nationality							0.002
Greek	397	94.50%	23	5.50%	420	100.00%	
Other	43	81.10%	10	18.90%	53	100.00%	
Education level							0.008
Elementary school–junior high school	17	85.00%	3	15.00%	20	100.00%	
Senior high school	122	93.10%	9	6.90%	131	100.00%	
University	173	95.60%	8	4.40%	181	100.00%	
Master’s degree–doctorate	71	98.60%	1	1.40%	72	100.00%	
Profession (Greek)							0.0001
Housewife	39	92.90%	3	7.10%	42	100.00%	
Unemployed	22	84.60%	4	15.40%	26	100.00%	
University student	8	80.00%	2	20.00%	10	100.00%	
Civil servant	114	98.30%	2	1.70%	116	100.00%	
Private employee	144	94.70%	8	5.30%	152	100.00%	
Self-employed	41	100.00%	0	0.00%	41	100.00%	
Other	15	88.20%	2	11.80%	17	100.00%	
Marital status							0.003
Married	27	75.00%	9	25.00%	36	100.00%	
Single	408	94.70%	23	5.30%	431	100.00%	
Separated	1	100.00%	0	0.00%	1	100.00%	
Divorced	6	85.70%	1	14.30%	7	100.00%	
Age							0.788
16–25	58	90.60%	6	9.40%	64	100.00%	
26–35	304	93.30%	22	6.70%	326	100.00%	
36–45	73	93.60%%	5	6.40%	78	100.00%	
Abortion							0.024
No	342	94.70%	19	5.30%	361	100.00%	
Yes	88	88.00%	12	12.00%	100	100.00%	
Partner’s agreement							0.003
No	7	63.60%	4	36.40%	11	100.00%	
Yes	427	94.50%	25	5.50%	452	100.00%	
Performed proposed tests							0.000
No	10	45.50%	12	54.50%	22	100.00%	
Yes	432	95.40%	21	4.60%	453	100.00%	

^1^ Derived from Fisher’s exact test.
